# Study concerning the design and functionality of individual emergency shelters

**DOI:** 10.1038/s41598-024-71327-0

**Published:** 2024-09-10

**Authors:** Mircea Costin Ene, Ionel Simion, Matei Valter

**Affiliations:** 1 Department of Graphic Engineering and Industrial Design, National University of Science and Technology POLITEHNICA Bucharest, 313 Splaiul Independenţei, 060042 Bucharest, Romania; 2 Aircraft Engineering with Pilot Studies, University of Salford, 43 Crescent, Salford, M5 4WT UK

**Keywords:** Homeless, Shelter, Emergencies, Engineering, Industrial engineering, CAD, CAE, Public health, Quality of life, Therapeutics, Civil engineering, Mechanical engineering, Materials for devices, Structural materials

## Abstract

The divide between the rich and poor in the European housing market is fast rising. Latest research indicates that Europe is dealing with an increasing number of homeless people. Every city in Europe has them—homeless people compelled to live on street corners, frequently hiding themselves with cardboard. Rain, snow, and temperatures below zero pose a threat to their lives on a daily basis. There are many varied kinds of services that have been discovered, but it is difficult to keep track of everyone and guarantee that they have a warm night's sleep in the winter. The current article suggests accommodation as a workaround until they can receive high-intensity support, a way to keep a single person warm and safe during the winter. The focus is on devising a strategy that not only ensures the warmth and safety of individuals during the harsh winter months but also seeks to industrialize the construction of shelters, ensuring affordability below the cost of winter hospitalization for a homeless person. Crucially, the article introduces an additional layer to this initiative by highlighting the dual purpose of these individual shelters. Beyond being a means to provide respite for the homeless during severe weather, these shelters are envisioned as immediate response units in the event of emergencies such as earthquakes in urban areas. The article explores the potential impact of this multi-layered approach on transforming urban landscapes and fostering resilient communities.

## Introduction

Despite Europe’s reputation for prosperity, boasting renowned destinations like the Riviera and prestigious Monte Carlo residences, it’s essential to acknowledge the harsh reality that millions of people across the continent are without shelter, grappling with homelessness in cities throughout the entire region.

Quantifying the homeless population poses significant challenges due to their transient lifestyle; often evading the attention of government and social service systems, some may even claim ownership over specific locations such as park benches or stairwells. While they may be familiar faces within local communities, they remain largely disconnected from the formal working world and its databases.

During cold seasons, these people seek cover in the warmest place they can find, whether a street corner or an abandoned building and because those places are hard to find, they usually suffer from frostbites and in many of the cases they have their limbs amputated.

But the hard truth is that they must face more than the bad weather, homeless people have to face their peers, the society that cannot understand the situation they are in. They often get beaten, have their goods stolen and are treated like less than a human.

In a decision of the European Parliament dated November 25, 2020, it was decided to stop the number of people ending up on the street without shelter, MEPs call on the EU and its member states to put an end to the lack of shelter for street people by 2030. They support an EU framework of national strategies and call on EU countries to decriminalize homelessness and continue to raise funds to tackle the problem^1^.

Housing is a fundamental human right, Parliament notes, but every night more than 700,000 people sleep rough in Europe, an increase of 70% in the last 10 years^2^. With the current economic downturn and job losses, the homelessness rate will increase even more.

European Parliament solutions for street people in the EU:

- ensure equal access to public services, such as healthcare, education and social services;
- supporting the integration of homeless people into the labor market through employment programs, training and adapted systems;
- ensuring constant access to emergency shelters of last resort (in addition to prevention and support measures);
- work on a common definition, better data collection and coherent indicators to be able to better understand and assess the extent of the problem.

Homeless persons should benefit from a warm bed, protection from the elements and other humans that might want to harm them. These shelters are only meant to be used during the colder months; they are not intended to take the place of the requirement for a permanent residence or a rental agreement that might provide a stronger sense of security and potentially a more balanced living^3^.

But moreover, these accommodations can be used as well in case of shelters, like earthquakes or floods for example. Authorities can use them as a quick response for the people who need a place to sleep until better solutions are implemented.

The fields of architecture and engineering hold a crucial role in the recovery efforts after disasters. Whether one is an engineer, architect, or designer, their impact is substantial in shaping the design and rebuilding of communities that have suffered from events like earthquakes and floods. In the complex process of recovery, these professionals become key players, using their skills and creativity to rebuild and strengthen communities. Their work goes beyond technical expertise; it becomes a beacon of hope, contributing to the renewal of areas affected by the challenges imposed by nature's forces. One of the most important roles is the design and construction of temporary or permanent housing that is safe, comfortable and resilient to future disasters. Post-disaster constructions are designed to help communities recover from a tragedy as quickly and efficiently as possible, such constructions are either temporary but quick-installation emergency shelters, or semi-permanent or permanent construction^4^.

The adaptability of these shelters makes them invaluable in emergency situations. In the event of any crises requiring rapid evacuation, these individual shelters can seamlessly transition into temporary emergency shelters. Equipped with the necessary facilities and resources, these shelters provide a rapid response to the immediate needs of displaced persons, ensuring that no one is left without a safe shelter at critical times.

## Relevance

The idea of individual accommodation is not a new one, but there is room for improvements and new ways of engineering the situation. Many others have implemented their innovative designs in order to do their part for the community and reach a hand for the ones in need. Kevin Passino, HEC director and professor of electrical and computer engineering at Ohio State, is among those focusing engineering solutions on humanitarian causes. Specifically, his textbook Humanitarian Engineering: advancing Technology for Sustainable Development contains several sections discussing homelessness and a range of technologies that can help. In this book he quotes

Technological advancement fosters prosperity in societies, yet it also disrupts existing structures, potentially undermining the economic advantages and political influence of specific groups. Sustainable economic growth relies on embracing new technologies and approaches, frequently led by newcomers.^5^.

Typical solutions range from creating low-cost and portable shelters, to providing technological solutions to provide lighting. However, much more is accomplished by finding more focused engineering solutions. This means really understanding the specific needs of homeless individuals and using smart engineering to create solutions that not only meet those needs but also help restore dignity and empowerment.

“You need to engineer things so it’s sort of people-proof,” Passino said in an interview. "Take candles for example—they’re light, cheap, and produce heat and light, but someone might roll over at night and kick it. A homeless woman told me one of her best friends burned to death.”^6^

**Fig. 1:**
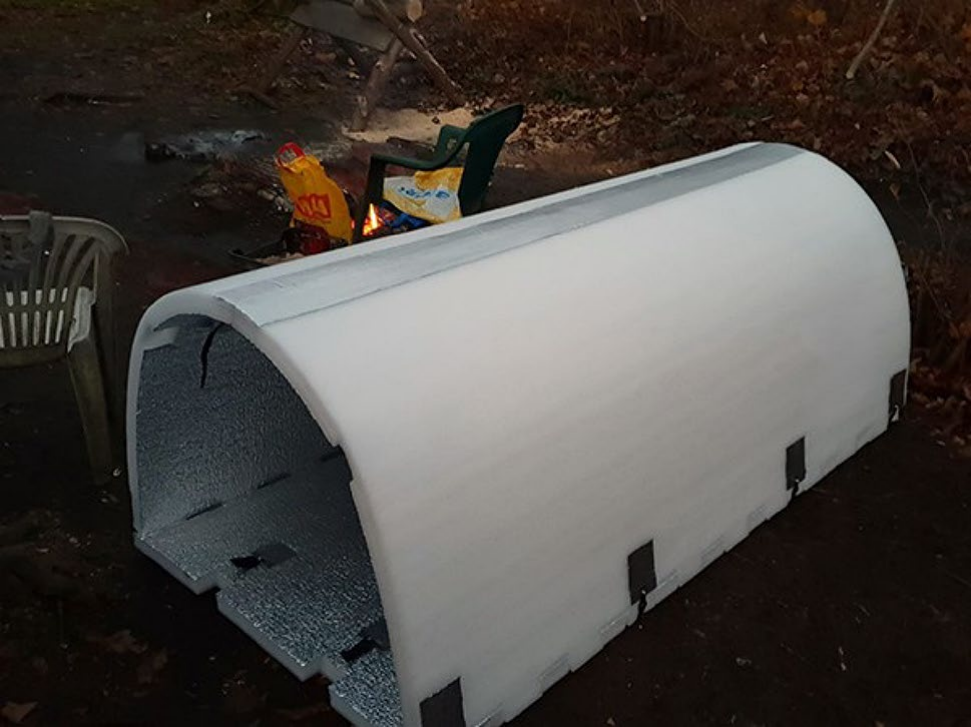
Ulmer nest.

A good example of people who have thought about the homeless in the streets and come up with a simple yet cost-efficient idea is Geoffroy de Reynal, a French engineer, who designed an igloo-like shelter from polyethylene foam, as seen in Fig. 1, a material that can retain body heat, with the inside covered in aluminum foil and the temperatures are estimated to be 15.5 °C higher than the outside^7^. These easy to carry shelters were given to the homeless people so they can set up a warm place for themselves to sleep wherever they want.

**Fig. 2:**
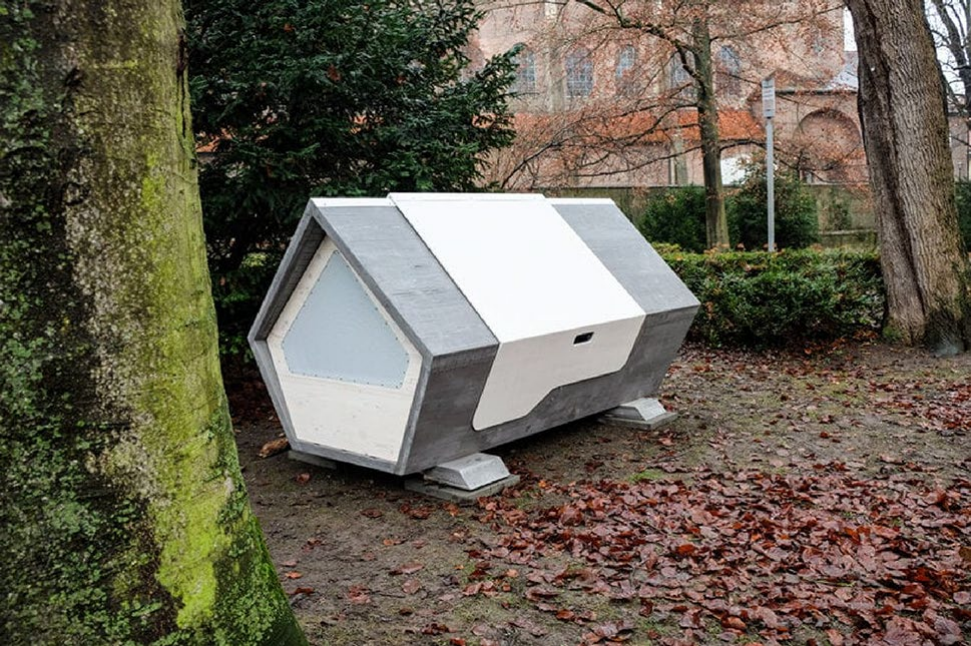
Igloo-like shelter.

Figure 2 showcases the Ulmer Nest, designed for individuals who, for various reasons, cannot access traditional social shelters. It’s important to note that the Ulmer Nest is not intended to replace standard social shelters but rather serves as a 'last resort' for those facing exposure to extreme weather conditions. The final prototype of the nest is constructed from solid wood, chosen for its durability, insulation properties, as well as its economic and ecological benefits. Additionally, powder-coated metal components facilitate intensive cleaning where necessary. Equipped with essential technologies such as a heat exchanger for fresh air, sensors, GPS, smoke alarms, and a motion detection system, the design prioritizes safety and functionality. The design is also fireproof and includes a secure locking system^8^.

These individuals have previously highlighted the issue and proposed innovative solutions aimed at saving lives and assisting public authorities in managing the situation more effectively. However, as the number of people facing homelessness continues to rise, it becomes increasingly challenging to quantify the extent of the problem.

Nonetheless, these initiatives serve as valuable provisional measures until more comprehensive solutions can be developed and implemented, predominantly in post-disaster scenarios. As Ashdown points out, 'Providing adequate shelter is one of the most intractable problems in international humanitarian response'^9^.

## Aim and objectives

**Fig. 3:**
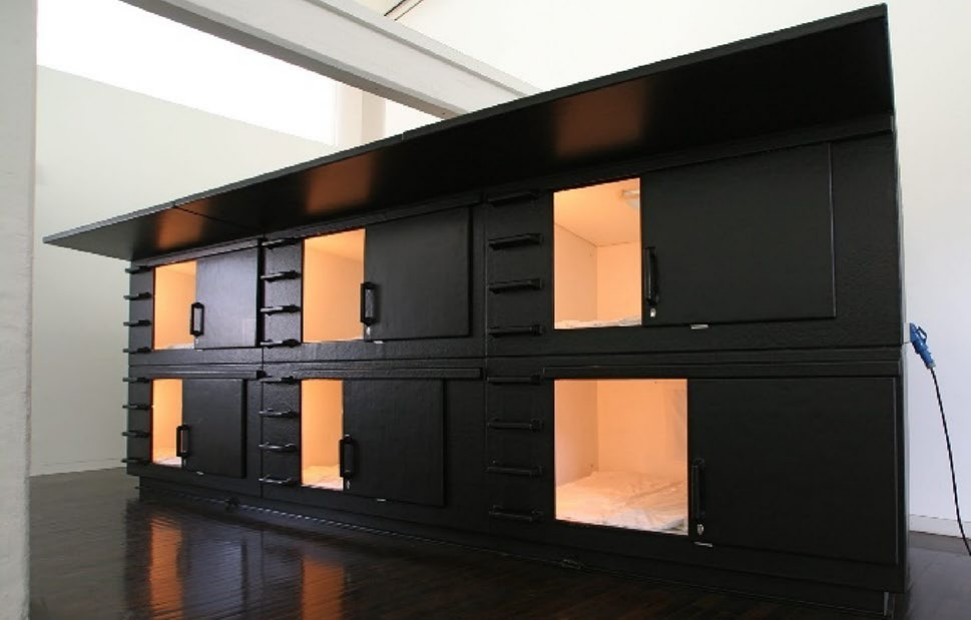
Mini-capsule hotel by Atelier Van Lieshout.

Taking these simple ideas and Atelier Van Lieshout design (Fig. 3) of their Mini-Capsule Hotel^10^, because of the way it uses the space efficiently, another design can be considered for the homeless population which the public authorities can manage in greater numbers.

Building upon the analysis of existing individual shelter designs, this project aims to synthesize the strengths and address the deficiencies identified in these innovative concepts. While the works of Passino, Reynal, and the Ulmer Nest team offer valuable insights, our approach focuses on overcoming the limitations observed in these designs. We emphasize affordability, simplicity in fabrication, and scalability.

To achieve affordability, the choice of materials and construction methods is carefully considered. By leveraging cost-effective yet durable materials, the shelter aims to balance resilience and economic feasibility. Additionally, the fabrication process is streamlined to minimize production expenses, making it suitable for mass production.

A key objective of this design is to reduce the number of homeless individuals requiring hospitalization due to complications caused by cold weather. This is particularly important for those who decline other options, such as social shelters, even when temperatures drop below 0 degrees Celsius. By preventing frostbite and associated complications like amputations—which often incur higher treatment costs than the investment in shelters

This shelter is designed to be assembled with minimal effort, ensuring that it can be rapidly deployed in emergency situations. Furthermore, its modular design facilitates ease of cleaning, maintenance and repairs, making it a sustainable and enduring solution.

In confronting the complex issue of homelessness, we recognize the need for innovative solutions.

## Research methods

Since the first time the concept of a smart city appeared, it has been the subject of increasing interest over the past few years. However, the vast majority of studies focus on upgrading a city’s infrastructure, including its government, transportation, communication networks, and information security and privacy. Social issues like poverty and homelessness are notably omitted from the topic of this research. This is another viable reason why we should think of these people more and realize that by helping them, we help our society grow and evolve at the same time, we can improve some aspects of their lives that we normally do not consider and do not realize how big of an impact it can have on them and the way they live their life^11^.

**Fig. 4:**
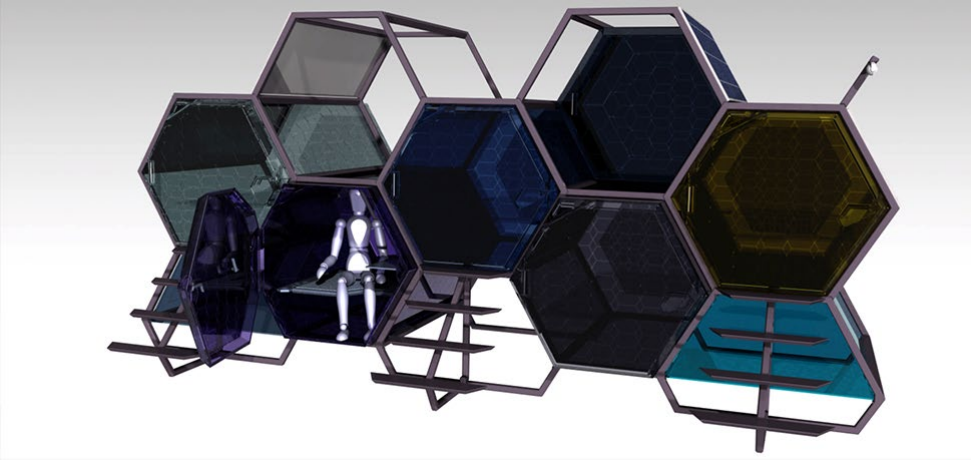
Whole assembly, modeled and rendered in CATIA V5. More information about CATIA V5 can be found at https://www.3ds.com/products-services/catia/.

Taking into account the previously provided examples, an additional method of constructing such a shelter is a single-person, plastic capsule that is staked one on top of the other like a honey bee comb to conserve as much space as possible as seen in Fig. 4. With this borrowed design from nature combined with the manufacturing technology available these days, we can offer them warmth and security in the winter and a safe place to sleep at night without having to worry about other people strolling along the street they have chosen to sleep on.

Moreover, the aspect of the shelter will affect the visual appearance of the surroundings; its design and colors must blend in and look like a modern solution for an older problem, it must show a new level of industrial evolution. Strategically located and well-designed shelters can improve the overall appearance of the city, transforming neglected spaces into areas that serve both functional and aesthetic purposes. This multi-functionality aligns with the principles of inclusive urban development and fosters a sense of community care. This is why many engineers and architects have tried some solutions to be integrated into city architecture and include art in the functional appearance of the product^12^.

The viability of the project lies in the structural and thermal tests conducted in CAE simulations, which assess the shelter’s ability to withstand dynamic loads and maintain acceptable internal temperatures under harsh conditions. This proposed design, rooted in practicality and informed by the successes and challenges of previous projects, aims to provide a reliable and innovative solution for those in need.

## Innovation and utility

**Fig. 5:**
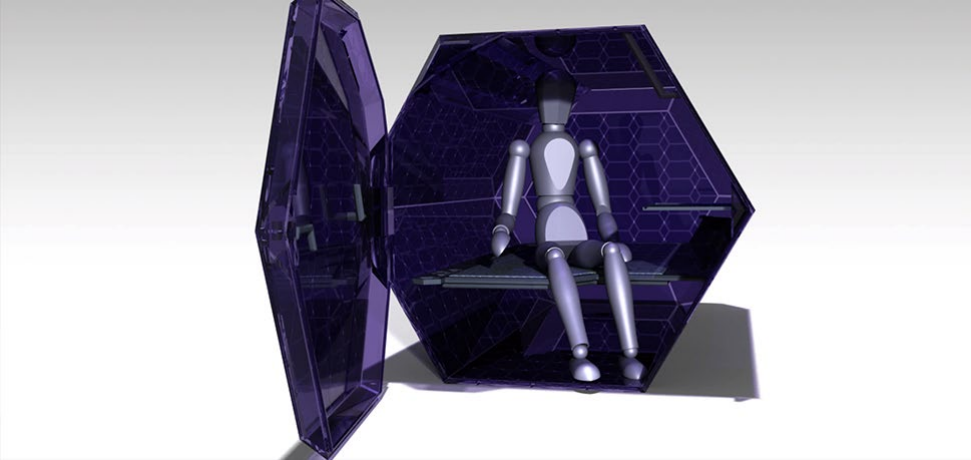
Capsule and Occupant, rendered in CATIA V5.

Five modular capsules as in Fig. 5, assembled as a unit, constructed from PMMA (polymethyl methacrylate) with an HDPE (high density polyethylene) frame.

These materials are commonly used in manufacturing due to their excellent properties, low cost, lightweight nature, and high level of recyclability. PMMA, also known as acrylic, offers shatter-resistant qualities, making it a safer alternative to glass in certain applications. For example, monolithic acrylic is often used for bulletproof glass. Additionally, acrylic is well-suited for injection molding, allowing it to be molded into virtually any shape. Its strength, combined with its ease of workability and machinability, makes it an ideal material for various consumer and commercial industries^13^.

Additionally*,* the translucence of PMMA enhances the sense of openness within the capsules, mitigating feelings of claustrophobia often associated with confined spaces. By allowing light to penetrate, PMMA creates a more spacious and comfortable environment for occupants.

**Fig. 6:**
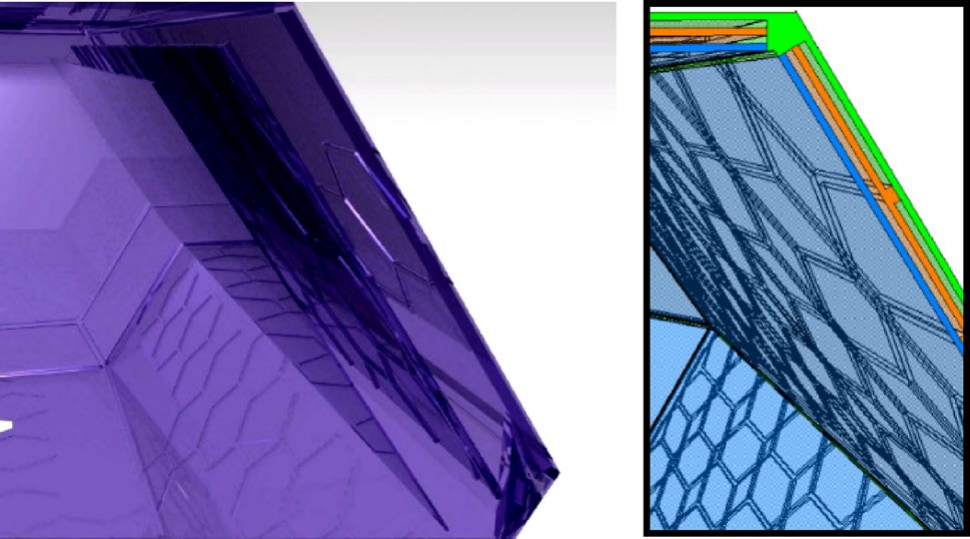
Wall installation and Section, created using CATIA V5.

The capsule itself is made from a single exterior shell, created through rotational molding, with added parts for its walls to strengthen them and create pockets of air to maintain the warmth within for a longer time. Figure 6 demonstrates how these walls can be mounted and how the air is enclosed within the hexagonal shapes.

The volume of air inside is approximately 2.9 m^3^, i.e., 2900 l of air. On average, a human occupies the equivalent of 66 l, which is subtracted from the total volume along with a piece of luggage, resulting in 2800 l. The amount of oxygen present in 2.8 cubic meters of air depends on the initial concentration of oxygen, which is usually around 21% at sea level.

Therefore, the amount of oxygen in 2.8 cubic meters of air would be:

1 $$ \left( {{2}.{8}00} \right) \, \left( {{21}/{1}00} \right) \, = {\text{ 588l}} $$

To calculate the number of minutes an individual can survive in a sealed container with 2.8 cubic meters of air at a rate of oxygen consumption of 0.5 l/min and carbon dioxide accumulation, the following formula can be used:

$$ {\text{Time }}\left( {{\text{minutes}}} \right) \, = \, \left( {\text{588l of oxygen}} \right) \, / \, \left( {{\text{oxygen consumption rate }}\left( {{\text{l}}/{\text{min}}} \right) \, + {\text{ carbon dioxide production rate }}\left( {{\text{l}}/{\text{min}}} \right)} \right) $$

The following assumptions will be used:

The oxygen consumption rate is 0.5 l/min. The carbon dioxide production rate is 0.5 l/min^14^.

Entering these values, the results will be:

2 $$ {\text{Time }}\left( {{\text{min}}} \right) = {588}/\left( {0.{5} + 0.{5}} \right) = {588}/{1} = {\text{588 min}} $$

Thus, the number of minutes an individual can survive in a sealed container of 2.8 cubic meters of air with an oxygen consumption rate of 0.5 l/min and an accumulation of carbon dioxide would be 588 min, close to 10 h. To ensure adequate ventilation, the capsule will be equipped with eight adjustable holes, which will facilitate the inflow of air. These holes will be operated by the occupant and serve to regulate the temperature inside the capsule.

**Fig. 7:**
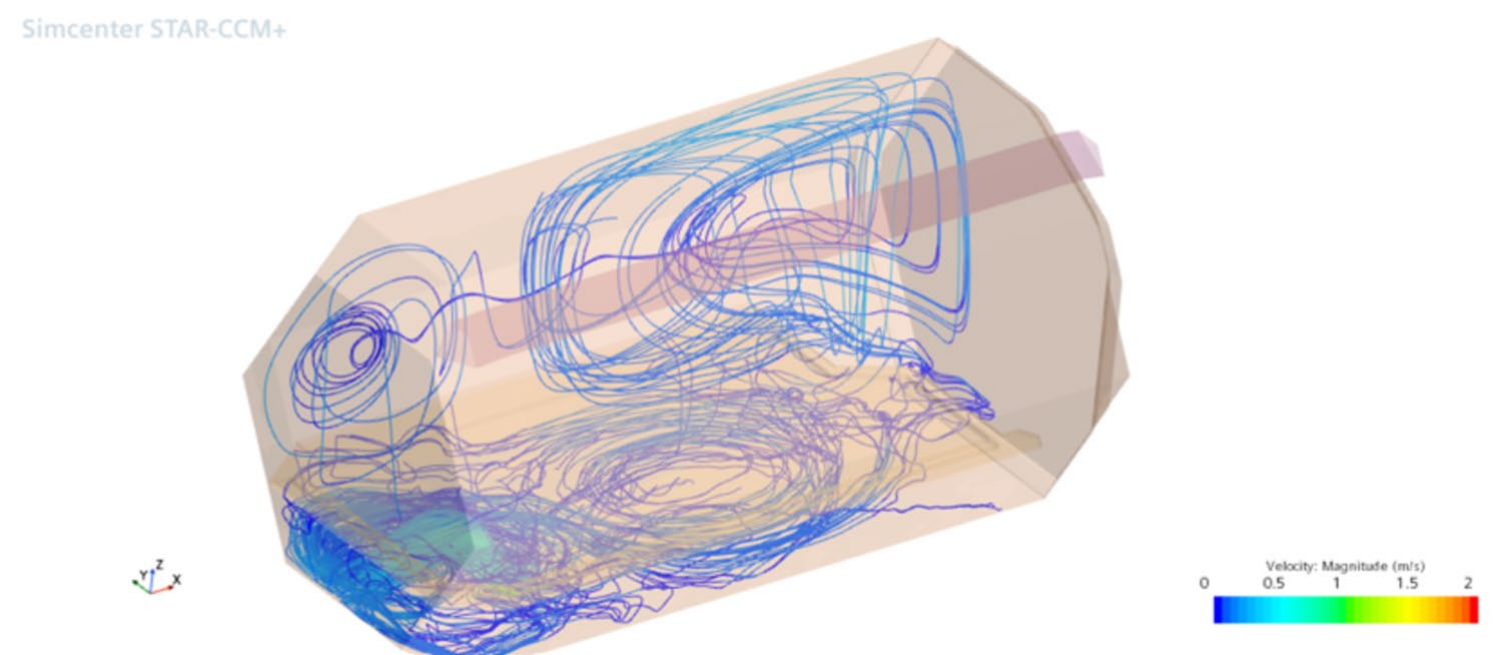
Inside airflow.

Additionally, a dedicated drainage hole, situated beneath the sleeping surface, will remain open at all times to serve as an effective outlet, particularly during shelter cleaning procedures. This feature also facilitates air circulation within the shelter, as demonstrated in the simulation depicted in Fig. 7.

The heating inside is provided by the bed surface, which operates similarly to a heated blanket with integrated electrical heating wires. This method has not been previously used in similar projects due to concerns about energy consumption. However, the dimensions of the assembly allow for the installation of enough solar panels on the dedicated frame and sufficient batteries in the service capsule to store the required energy. Simulations have shown that under optimal operating conditions, even with direct, high, and cold wind blowing into the capsules, it is possible to maintain a livable temperature inside.

Several tests were conducted across a spectrum of temperatures, with a predominant focus on sub-zero conditions, aimed at observing the temperature dynamics within the individual shelters. The objective was to simulate different temperature exposure that could be applied to real-life situations, providing a foundational understanding of the shelter's performance across varying climatic scenarios.

It is important to note that for optimal results, the heated bed must be turned on at maximum capacity, and additionally, the ventilation inside must be closed and only the drain hole underneath the capsule will remain open.

Table 1 contains the input data used for the thermal simulations for a capsule and an occupant inside.

**Table 1:** Input.

	Temp − 8 °C	Temp − 15 °C	Temp − 18 °C
Wind speed	12 m/s	12 m/s	12 m/s
Wind direction	Longitudinal	Longitudinal	Longitudinal
Mannequin	33 °C	33 °C	33 °C
Bed temp	56 °C	56 °C	56 °C
Ground temp	− 2 °C	− 2 °C	− 2 °C

In the initial test, conducted at – 8 °C—falling below the average temperature of – 5 °C recorded in January in Bucharest^15^—the presence of a heated bed and the well-insulated walls, produced a notable elevation in the internal temperature, reaching up to a commendable 21 °C. This observation underscores the efficacy of the shelter's heating mechanism, particularly in conditions mirroring or exceeding the typical winter average temperatures in the region.

In the second test, the average temperature in February in Greenland^15^, one of the coldest countries in Europe with an average temperature of – 15 °C, and even in temperatures as low as this, the inside temperature reached 16 °C.

**Fig. 8:**
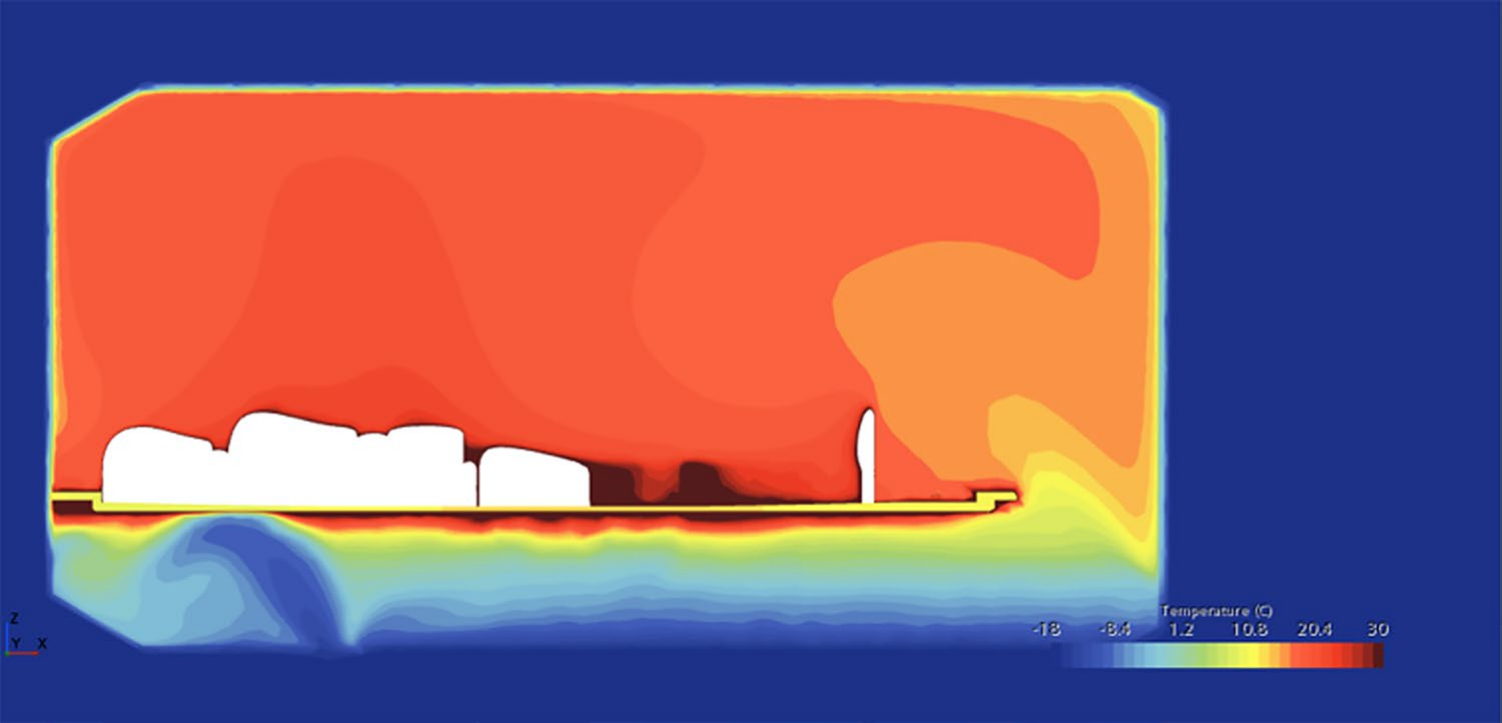
− 18 °C simulation.

The third and most severe test was conducted at − 18 °C, a temperature commonly used in car manufacturers' defrosting tests. Remarkably, even under such extreme conditions, the internal temperature within the shelter reached a noteworthy 14.3 °C. As shown in Fig. 8, this significant evidence not only underscores the shelter's robust thermal performance but also positions it as a viable solution for harsh winter climates.

Such important observations form a crucial foundation for the academic exploration of the shelter's thermal dynamics, offering valuable considerations for their real-world implementation across diverse climatic contexts.

**Fig. 9:**
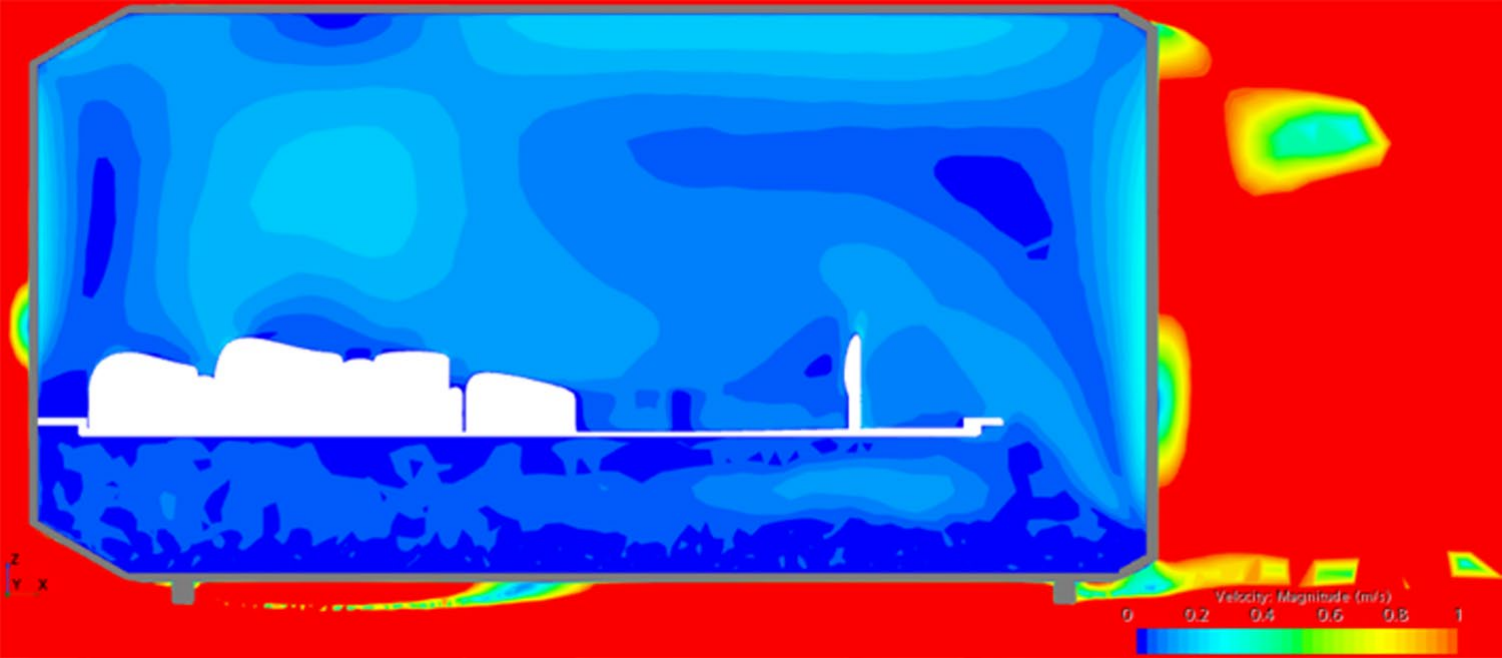
Air circulation—temperature gradient.

In these simulations, both the airflow inside and outside the capsule (Fig. 9), as well as the pressure differential caused by temperature variations and wind speed, were observed. The simulations consistently showed normal pressure values, indicating no harm to the occupants. Table 2 summarizes the results.

**Table 2:** Results.

	Av. temp. inside (°C)	Max temp walls (°C)	Min. temp walls (°C)	Av. air speed inside
Temp − 8 °C	21.1	56	− 8	0.084
Temp − 15 °C	16.1	56	− 15	0.083
Temp − 18 °C	14.3	56	− 18	0.086

**Fig. 10:**
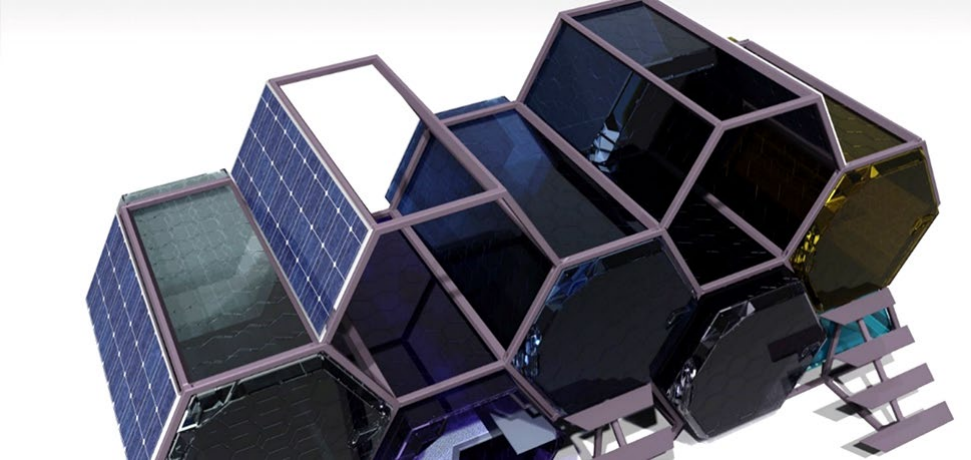
Solar panels configuration.

To ensure streamlined future maintenance by public authorities, it is imperative to restrict the use of electronic components within the shelter framework. Besides the previously mentioned heating source, the occupant is provided with limited electronic amenities, such as a light source and a plug for phone recharging. If the assembly cannot be directly connected to the grid to provide energy for each individual capsule, a supplementary framework can be installed on top of the structure, as shown in Fig. 10.

This added framework serves an important role by incorporating at least four solar panels and a service capsule. The service capsule is specifically designed to store batteries and essential components needed for energy storage and distribution to individual shelters. This comprehensive integration ensures the establishment of a self-sustaining configuration. The inclusion of these strategic components positions the shelter as a model of energy efficiency and self-sufficiency in the realm of emergency housing solutions.

## Structural tests

**Fig. 11:**
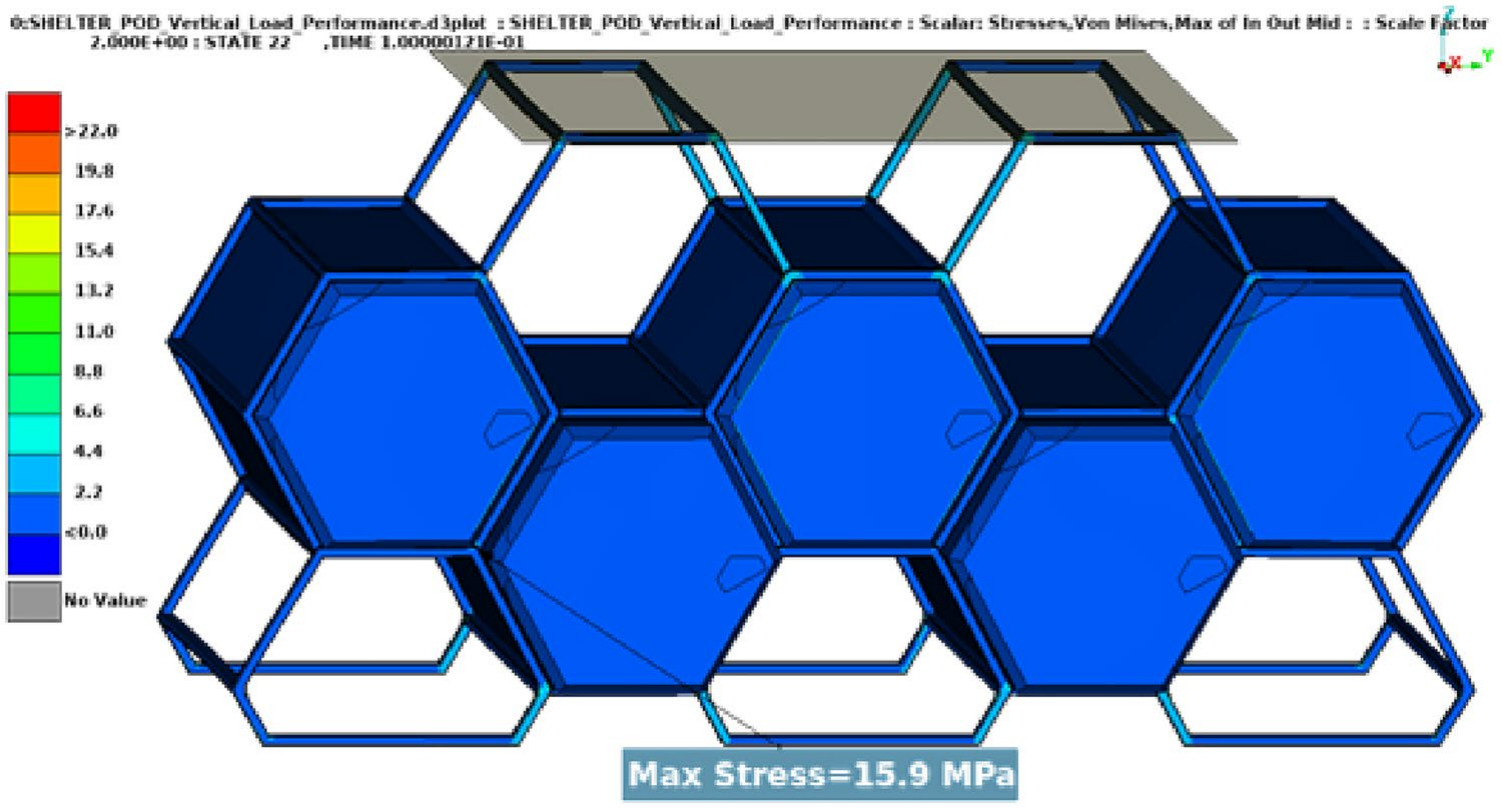
Results vertical load.

**Fig. 12:**
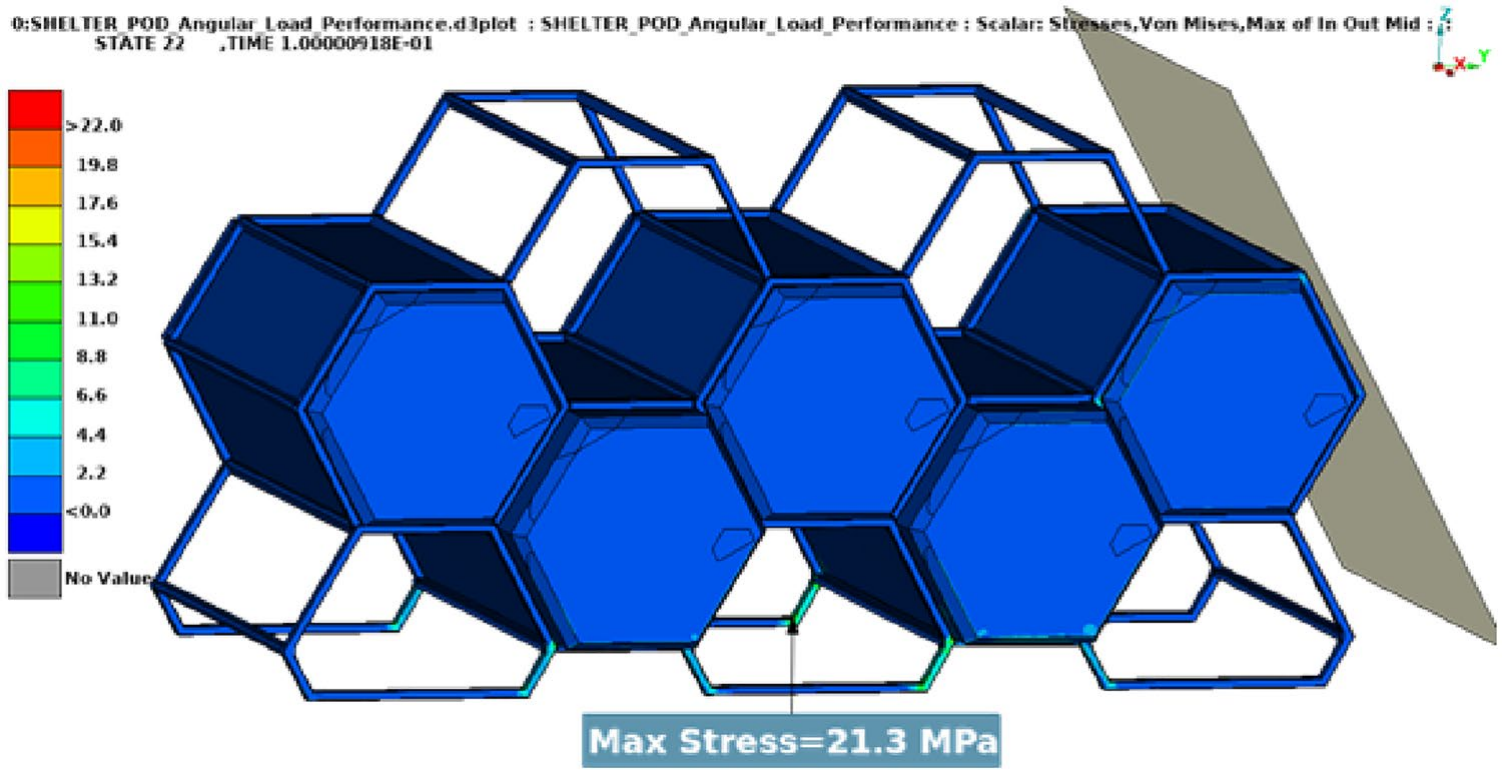
Results angular load.

In these tests, two types of dynamic loads were applied to the entire assembly, one vertical load equal to the maximum weight of the assembly when it is fully occupied (Fig. 11), close to 31,882.5 N and one angular load of 24,525 N, close to 2.5 tons (Fig. 12).

**Fig. 13:**
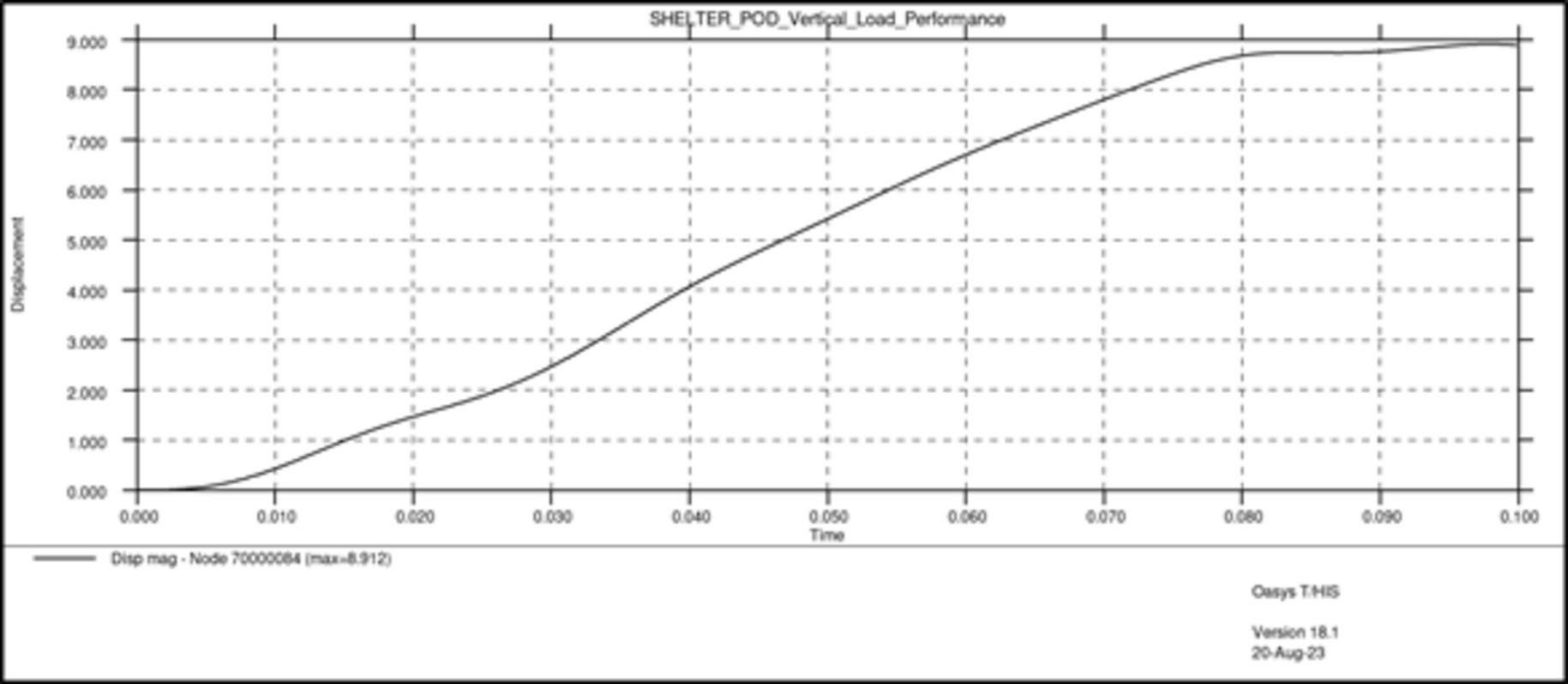
Graphic vertical load stress.

**Fig. 14:**
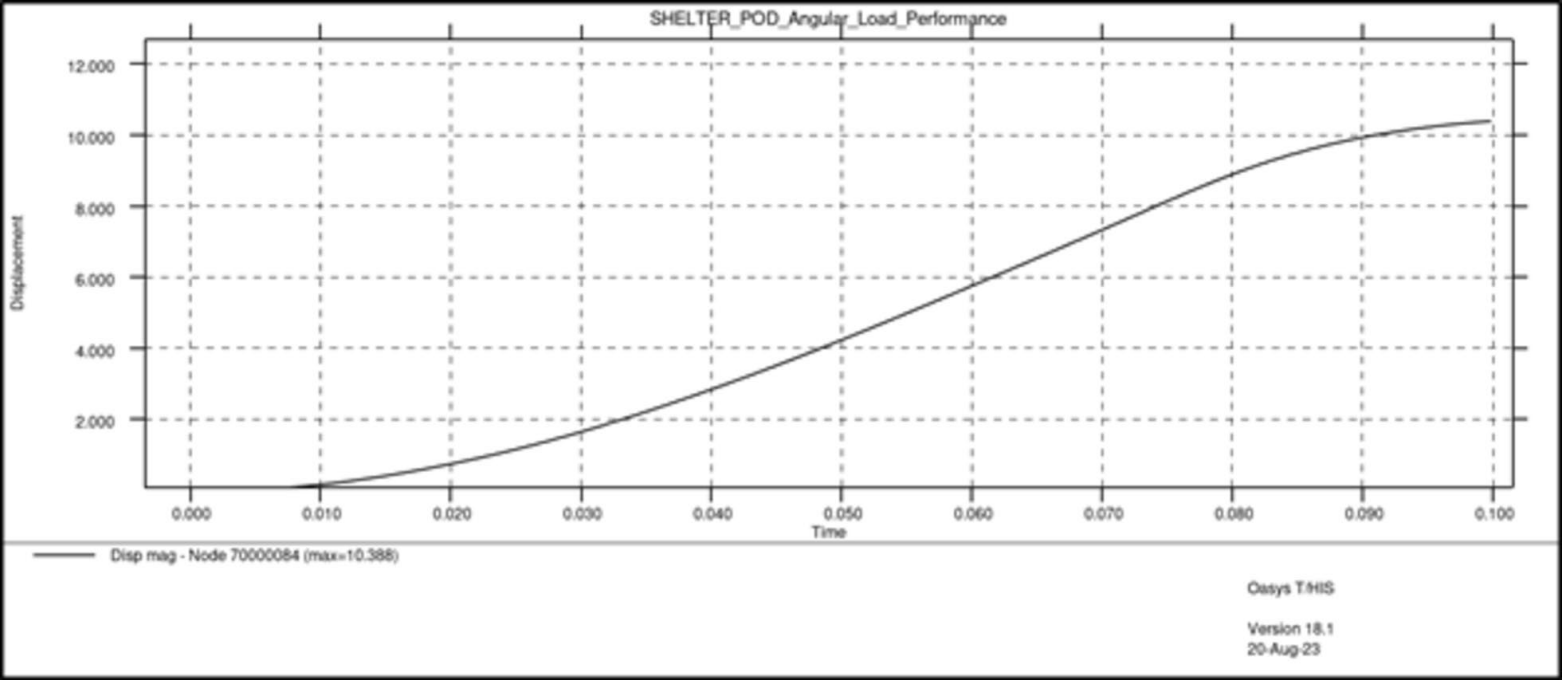
Graphic angular load stress.

Both load cases were constructed using a rigid plane that will push the structure with a specific load. The plane is also fixed in all directions except the one on which the load is applied. The type of load used is dynamic because a dynamic load, even with the same weight, is more damaging than a static one. Dynamic loads involve movement and changing forces that can cause greater stress and strain on materials and structures compared to stationary loads (Figs. 13, 14).

**Fig. 15:**
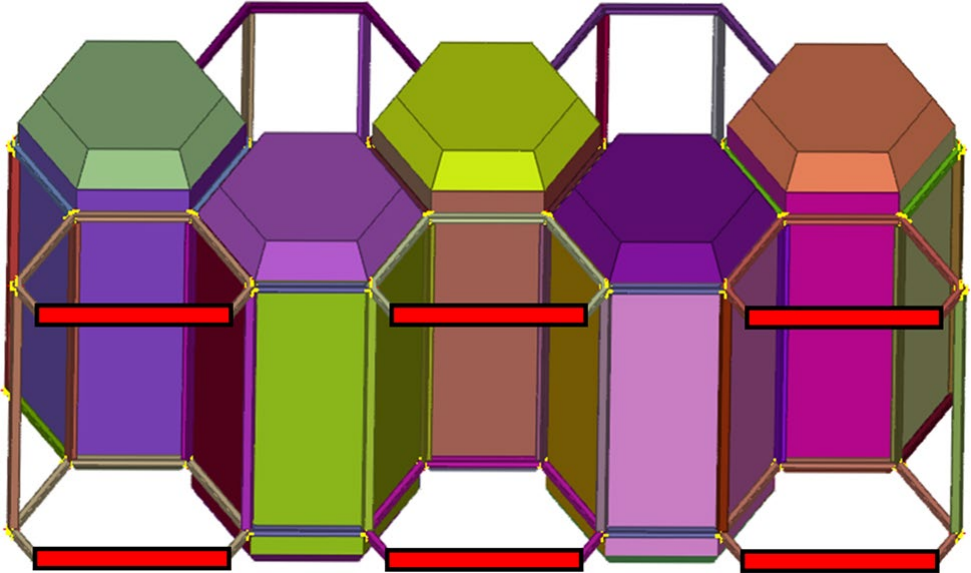
Contact surfaces.

In the CAE model, the area that would be in contact with the ground was modeled as a rigid part with locked translation and rotation (Fig. 15).

The contact force between the impactor plane and the structure was plotted. Due to the deformations that occur in the model during running, some fluctuations in the contact force are expected to be observed, although the force applied to the impactor had a linear increase and a plateau.

The aim was to ensure that the required force is applied to the model and that a stabilization of the force is observed towards the end of the simulation.

**Fig. 16:**
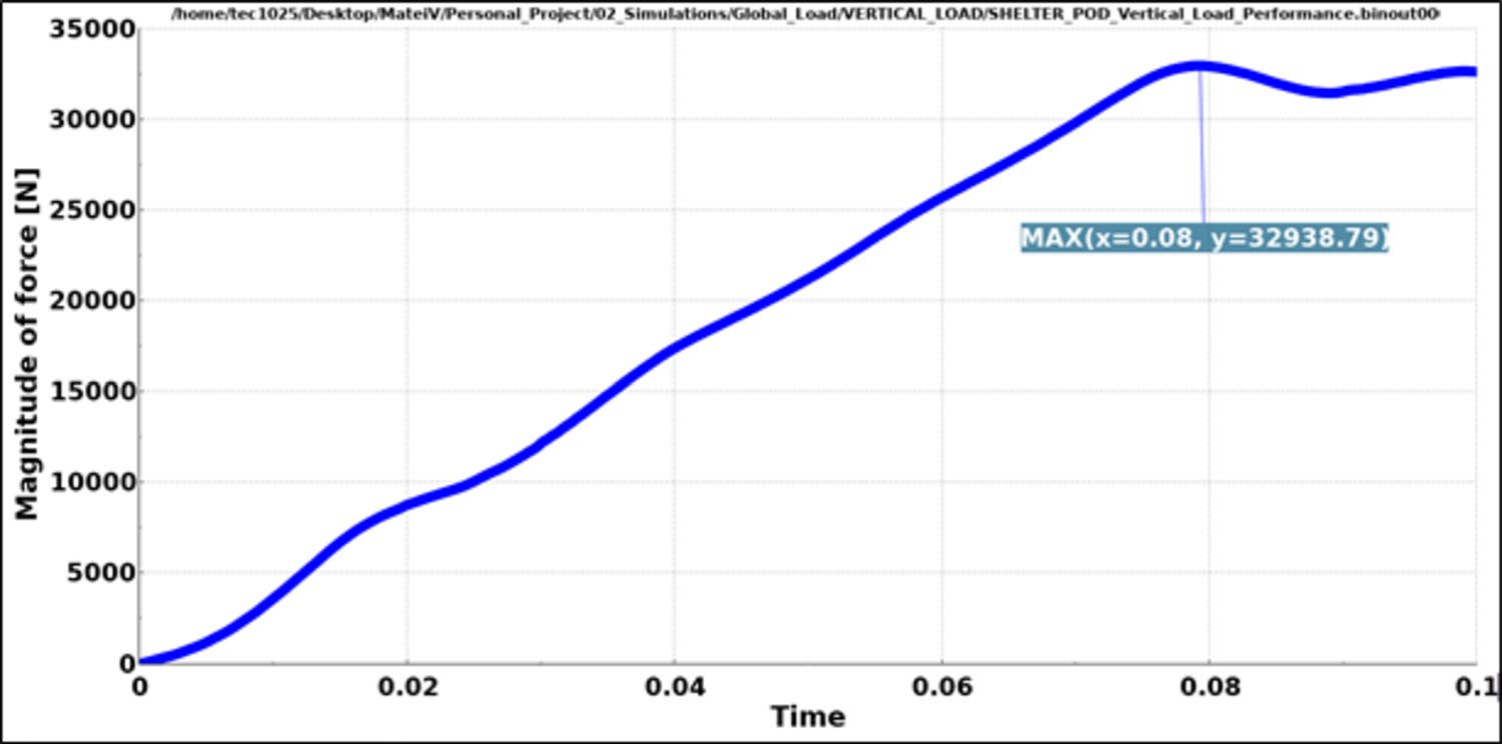
Magnitude of vertical load.

**Fig. 17:**
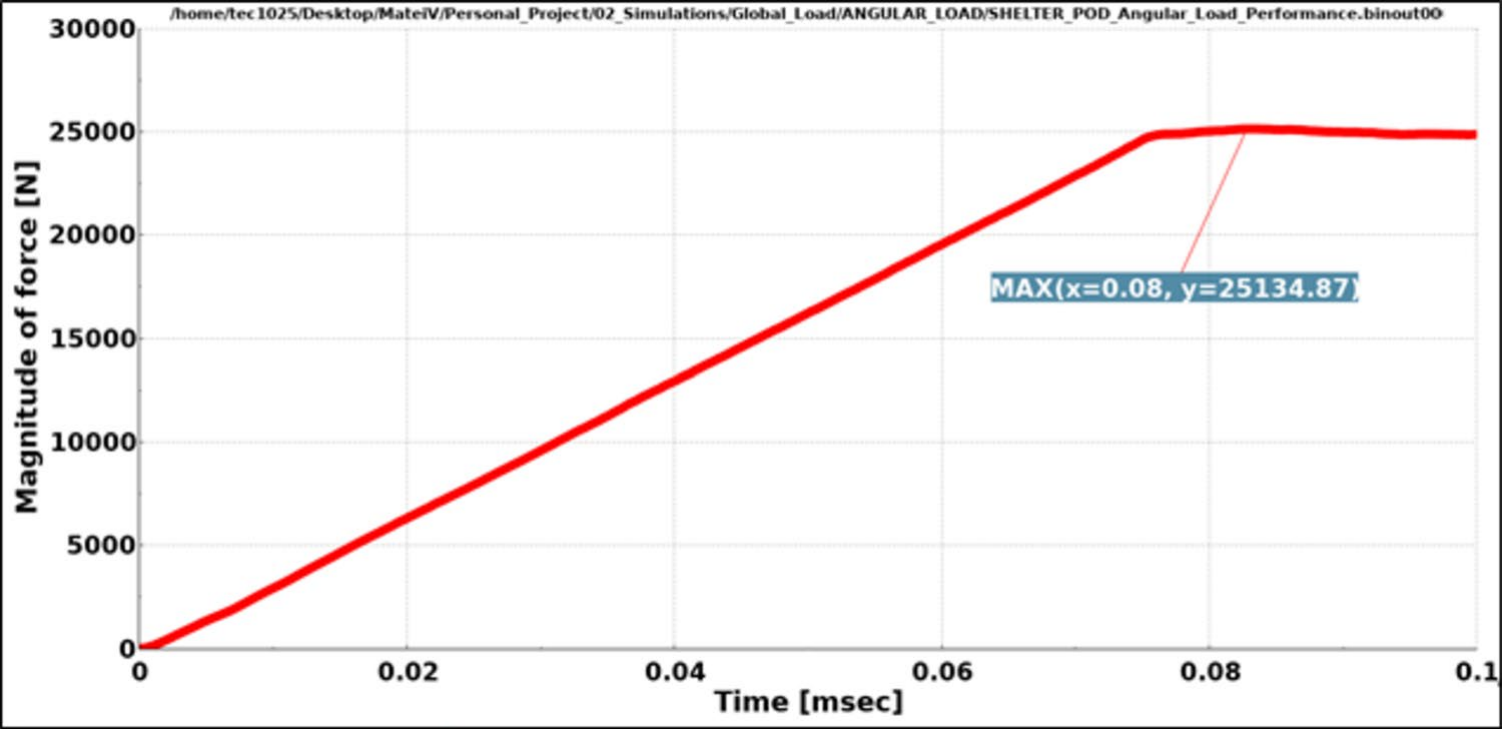
Magnitude of angular load.

In both cases, Figs. 16 and 17, even though fluctuations are observed, we see that the expected load is achieved even with some peaks which are higher.

Results have shown very good overall performance in the structural tests. While in the vertical load case the impactor plane only moved 8.9 mm (Fig. 13), the angular test is evidently more severe since the rigid plane moved 10.4 mm (Fig. 14). However, the structure can still withstand the load applied. A safety factor was included in the calculations while using the angular load. Each capsule was designed to hold a 150 kg mass consisting of a person and their belongings. This projected weight is significantly more than what would be expected in a real-world scenario, ensuring that the material remains comfortably within its plastic limit.

These tests were also necessary to ensure that the frame can be hollow inside, with a 10 mm wall thickness to facilitate the routing of the cables to each capsule.

Both structural and thermal simulations have demonstrated the assembly's viability and efficiency. Alongside the ease of production, installation, and maintenance, the assembly can be considered an improved multi-layered solution.

## Transportation and deployment

In order to be used as a rapid disaster response, the capsules must be able to be quickly loaded, transported, and deployed where needed. An open platform truck is an ideal transport solution for oversized containers, accommodating dimensions of 3 m high, 3.2 m wide, and 12 m long^16^.

These trucks can carry large and heavy containers in one shipment, as they are authorized for oversized transport. This can significantly reduce shipping costs and make delivery more affordable.

**Fig. 18:**
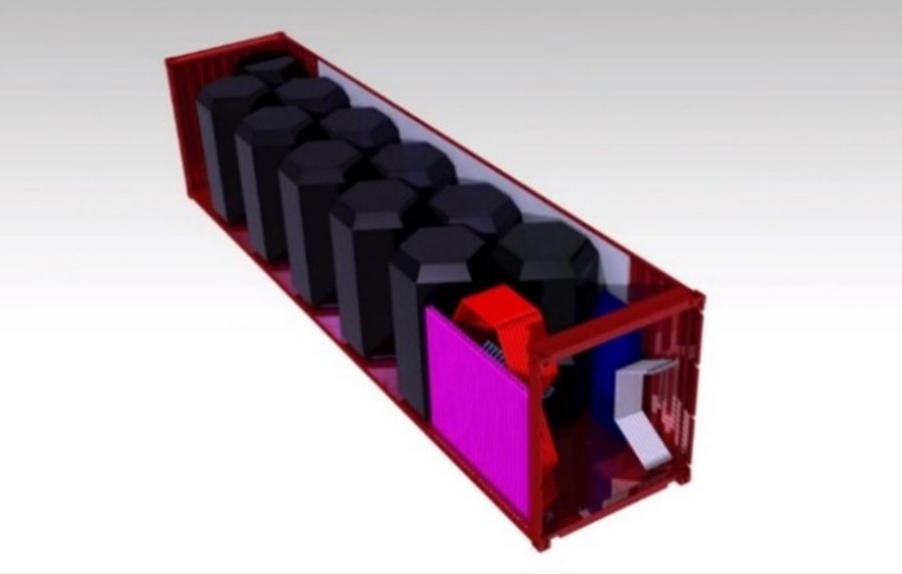
Loaded container.

Therefore, considering the dimensions and the design of the assembly, the most recommended option is the use of the 12 m container, which allows for the transportation of up to 2 assemblies. This equates to 10 capsules along with additional equipment. Figure 18 illustrates how this can be achieved. Unfortunately, the container can only accommodate either 2 assemblies with the necessary equipment for energy independence or the extra storage space.

**Fig. 19:**
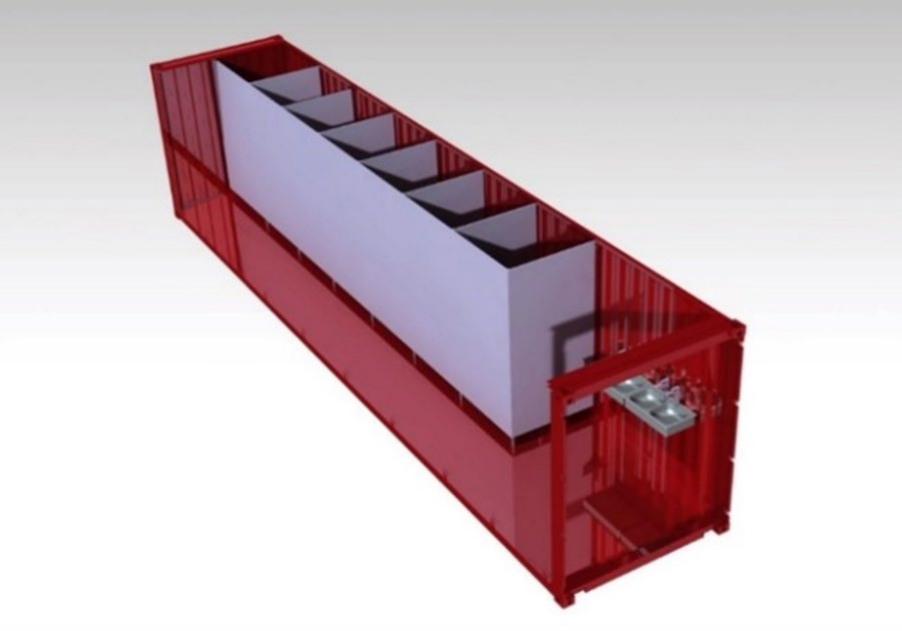
Transformed container.

After unloading and installing the two assemblies, the container can be transformed into a fully functional sanitary unit, complete with showers and toilets, by unfolding the pliable walls pre-installed in the container. One of the possible designs can be seen in Fig. 19. This adaptive repurposing of containers for emergency sanitation not only provides essential facilities but also offers considerable advantages in promptly meeting urgent needs in disaster-affected regions, ensuring swift and effective response efforts.

## Conclusions

The principal objective of the project was to enhance affordability for public authorities, ensuring sustained and manageable upkeep. The design facilitates uncomplicated storage during warmer seasons, reserving deployment for colder seasons or emergencies. Internal electronic components emphasize ease of replacement in the event of malfunction. The production process has been simplified to facilitate efficient, large-scale manufacturing, especially for regions with high homelessness rates. These shelters are as easy to clean as existing public facilities, ensuring practicality in maintenance and hygiene. Additionally, social workers can easily locate and access the shelters to provide necessary support and supplies.

Simulations have shown that the heating source, combined with a well-insulated capsule, can raise the interior temperature to a comfortable level, significantly reducing exposure to harsh weather.

While we acknowledge that, like the other solution presented in this paper, the individual shelter may not offer an absolute guarantee of survivability, it represents a significant improvement over the plain reality faced by those sleeping on city streets during cold seasons.

In summary, the presented project effectively addresses legislative gaps and engineering needs while excelling in safety considerations. By combining legislative advocacy with innovative yet simple engineering design, these shelters offer a promising solution to improve the living conditions of those in need.

## Discussions

For the legislative impact on the project, the conditions and description of the project pertain to an unregulated area, which aims to provide modular housing for disadvantaged people. We consider that the project is viable but requires a close partnership between the Ministry of Labor and Social Protection, the Directorate for the Protection of Persons with Disabilities, the Directorate for Social Inclusion, the County Council, as appropriate.

The effort to implement the project from a legislative perspective is to initiate a dialogue with the authorities to point out that the current system does not support some disadvantaged people for operational reasons, and the purpose of the project is to provide night shelters that meet the legislative standards imposed by current regulations.

The legislative framework also permits the utilization of the proposed solution in emergency scenarios, such as seismic events like earthquakes or floods. According to a report by the Central Disaster Prevention Council (CDPC), following the Niigata Earthquake in Japan, the number of evacuees in shelters peaked at over 100,000 within four days. By the end of the first month, this number had decreased to about 10,000 as people found alternative housing solutions. This emphasize the necessity of readiness in post-disaster situations, highlighting the importance of these shelters in providing immediate help until more permanent accommodations can be secured^17,18^.

To emphasize all of the above, a prototype needs to be implemented and tested in real-life situations, and the resulting data should be compared with the existing data from the simulations.

## Data Availability

The datasets generated and supporting the findings of this article are obtainable from the corresponding author upon reasonable request.
